# Effect of skilled reaching training and enriched environment on generation of oligodendrocytes in the adult sensorimotor cortex and corpus callosum

**DOI:** 10.1186/s12868-017-0347-2

**Published:** 2017-03-09

**Authors:** Silke Keiner, Fanny Niv, Susanne Neumann, Tanja Steinbach, Christian Schmeer, Katrin Hornung, Yvonne Schlenker, Martin Förster, Otto W. Witte, Christoph Redecker

**Affiliations:** 1 Hans Berger Department of Neurology, Jena University Hospital, Am Klinikum 1, 07747 Jena, Germany; 2 Department of Cardiothoracic Surgery, Jena University Hospital, Am Klinikum 1, 07747 Jena, Germany; 3 Pneumology, Internal Medicine I, Jena University Hospital, Am Klinikum 1, 07747 Jena, Germany

**Keywords:** NG2 cells, Sensorimotor activity, CNPase, Doublecortin, GSTπ

## Abstract

**Background:**

Increased motor activity or social interactions through enriched environment are strong stimulators of grey and white matter plasticity in the adult rodent brain. In the present study we evaluated whether specific reaching training of the dominant forelimb (RT) and stimulation of unspecific motor activity through enriched environment (EE) influence the generation of distinct oligodendrocyte subpopulations in the sensorimotor cortex and corpus callosum of the adult rat brain. Animals were placed in three different housing conditions: one group was transferred to an EE, a second group received daily RT, whereas a third group remained in the standard cage. Bromodeoxyuridine (BrdU) was applied at days 2–6 after start of experiments and animals were allowed to survive for 10 and 42 days.

**Results:**

Enriched environment and daily reaching training of the dominant forelimb significantly increased the number of newly differentiated GSTπ^+^ oligodendrocytes at day 10 and newly differentiated CNPase^+^ oligodendrocytes in the sensorimotor cortex at day 42. The myelin level as measured by CNPase expression was increased in the frontal cortex at day 42. Distribution of newly differentiated NG2^+^ subpopulations changed between 10 and 42 days with an increase of GSTπ^+^ subtypes and a decrease of NG2^+^ cells in the sensorimotor cortex and corpus callosum. Analysis of neuronal marker doublecortin (DCX) showed that more than half of NG2^+^ cells express DCX in the cortex. The number of new DCX^+^NG2^+^ cells was reduced by EE at day 10.

**Conclusions:**

Our results indicate for the first time that specific and unspecific motor training conditions differentially alter the process of differentiation from oligodendrocyte subpopulations, in particular NG2^+^DCX^+^ cells, in the sensorimotor cortex and corpus callosum.

**Electronic supplementary material:**

The online version of this article (doi:10.1186/s12868-017-0347-2) contains supplementary material, which is available to authorized users.

## Background

The adult brain responds in a remarkable way to physical exercise, enriched environment and motor learning paradigms via morphological, cellular and functional changes [22, 24, 25, 27, 32, 54]. Several studies have reported that learning and motor activity influence oligodendrocytes and myelination in white and grey matter of the adult brain [8, 12, 13, 29, 35, 52, 55, 56]. In particular, enriched environment increases myelin-forming oligodendrocytes, myelinated axons and white matter volume during brain aging [47, 48, 57, 59]. Furthermore, McKenzie et al. [35] demonstrated that wheel running remarkably increases the proliferation and generation of oligodendrocytes in the corpus callosum (CC). These newly differentiated oligodendrocytes are necessary to learn complex running wheel.

Previous studies have shown that oligodendrocyte precursor cells such as chondroitin sulphate proteoglycan expressing cells (NG2^+^ cells) react to learning and motor activity in the adult brain [5–7, 9, 31, 35, 36, 41, 46, 55]. A small fraction of proliferating NG2^+^ cells differentiate into mature oligodendrocytes [4, 11, 21, 37]. In addition, previous studies demonstrated that NG2^+^ cells are able to express the neuronal marker doublecortin (DCX), a microtubulin-associated protein [19, 20]. DCX is known as a key player in neuronal migration during development. Oligodendrocyte precursor cells expressing NG2 are also known to be highly migratory cells in vitro and in vivo [21, 33]. In the adult brain, a subpopulation of NG2^+^cells can also express DCX which is involved in microtubulin polymerization [33]. However, in which way reaching training and enriched environment influences generation of distinct oligodendrocyte subpopulations and whether NG2-cells respond in the adult cortex and corpus callosum, regions where oligodendrocytes are present, is still unclear.

The aim of the present study was: (1) to evaluate the effect of motor activity on generation and distribution of different oligodendrocytes subtypes in the grey and white matter of the adult rat brain and (2) to examine co-expression of DCX in NG2^+^ cells and to what extent RT and EE alter the differentiation of NG2^+^DCX^+^ subpopulations.

We wanted to test the hypothesis that specific reaching training is able to influence the differentiation of specific oligodendrocyte subpopulations in the grey and white matter, as compared to unspecific motor training by enriched environment.

## Results

### Effect of RT and motor training by EE on brain volume

**Fig. 1:**
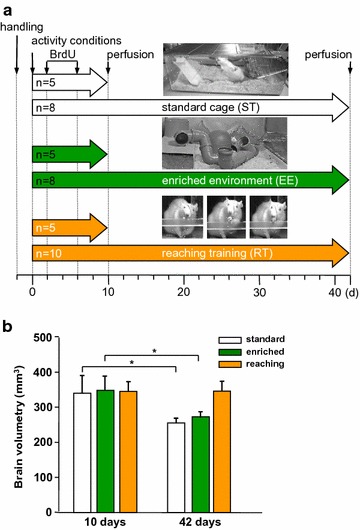
**a** Experimental design: three different groups of animals were housed under standard conditions in ordinary cages (standard), in an enriched environment (enriched) containing various objects such as horizontal and vertical boards, climbing platform, plastic tubes and tunnels, chains, and small houses or kept in a standard cage and trained daily in a reaching task (reaching) of the dominant forelimb in a Plexiglas chamber. Animals had to grasp 50 food pellets in the first week, which was raised up to 100 pellets per day in the second week. All animals received single daily i.p. BrdU injections from day 2 to day 6. Animals were transcardially perfused for further processing at day 10 or day 42 after the beginning of the experiments. **b** The graph represents the brain volume of different groups for 2.7 to −5.3 mm refered from Bregma. *Bars* represent mean ± SD. *Asterisks* indicate significant differences (*P* < 0.05)

On day 10, analysis of global brain volume (Bregma 2.7–5.3 mm) revealed no differences between the 3 experimental groups (standard: 349 ± 52 mm^3^, enriched: 357 ± 42 mm^3^, reaching: 354 ± 29 mm^3^). Interestingly, brain volumes were significantly reduced in the standard group (−25%, *P* < 0.001) as well as in the enriched group between day 10 and day 42 (−22%, *P* < 0.001), whereas no loss of brain volume was found in the reaching group (standard: 262 ± 14 mm^3^, enriched: 280 ± 16 mm^3^, reaching: 355 ± 29 mm^3^) at day 42 (Fig. 1b).

### NG2^+^ and GSTπ^+^ expressing oligodendrocytes in the sensorimotor cortex

BrdU^+^ cells were observed in all cortical layers without any laminar preference, in all experimental groups. EE significantly decreased the number of BrdU^+^ cells in the sensorimotor cortex at day 10 compared to standard housing and RT. No differences were observed at day 42 (Additional file 1: Table S1).

In addition, proliferating NG2^+^ cells were quantified after EE and RT and compared to standard housing at day 10 and 42. We found no change in the total number of NG2^+^ cells in either activity group, but newly differentiated NG2^+^ cells were significantly reduced after EE housing at day 10 compared to standard housing conditions (*P* = 0.029). No effects were observed in the RT group (Additional file 1: Table S1).

**Fig. 2:**
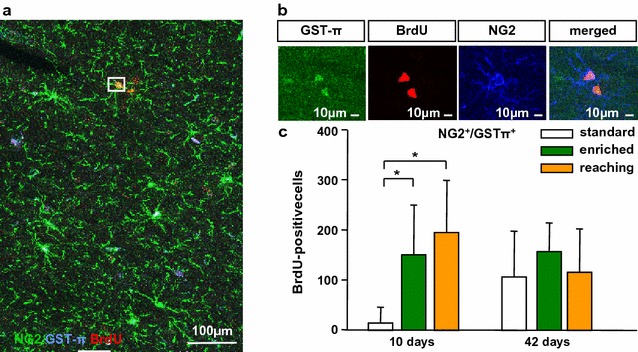
Activity-dependent increase of BrdU^+^GSTπ^+^ cells in the sensorimotor cortex at day 10. **a** Confocal images of triple-labelled sections with antibodies against BrdU (*red*), NG2 (*green*) and GSTπ (*blue*). **b** Single confocal planes by z-stack co-labelling of BrdU^+^ with NG2^+^GSTπ^+^. **c** Graph depicting the total number of BrdU^+^NG2^+^GSTπ^+^ cells which increased after enriched environment housing and reaching training at 10 days compared to standard conditions. *Bars* represent mean ± SD. *Asterisks* indicate significant differences (*P* < 0.05). Scale bars represent **a** 100 µm and **b** 10 µm

Furthermore, we analysed the influence of EE and RT on newly differentiated NG2^+^GSTπ^+^ cells in the sensorimotor cortex. A small number of BrdU^+^NG2^+^GSTπ^+^ cells was observed in all cortical layers without any laminar preference in all experimental groups (Fig. 2a). 3D-reconstructions with laser scanning microscopy showed a high overlapping of NG2 and GSTπ expression with BrdU^+^ cells (Fig. 2b). Both RT (*P* = 0.032) and EE (*P* = 0.032) housing transiently increased the number of BrdU^+^NG2^+^GSTπ^+^ oligodendrocytes at day 10 compared to the standard group (Fig. 2c). In addition, we found a motor activity-dependent increase in the number of newly differentiated NG2^+^GSTπ^+^ oligodendrocyte precursor cells in the sensorimotor cortex at day 10. Interestingly, the percentage of BrdU^+^NG2^+^GSTπ^+^ cells was increased compared to standard housing at day 10 (Additional file 2: Fig. S1).

### CNPase^+^ oligodendrocytes in the sensorimotor cortex

**Fig. 3:**
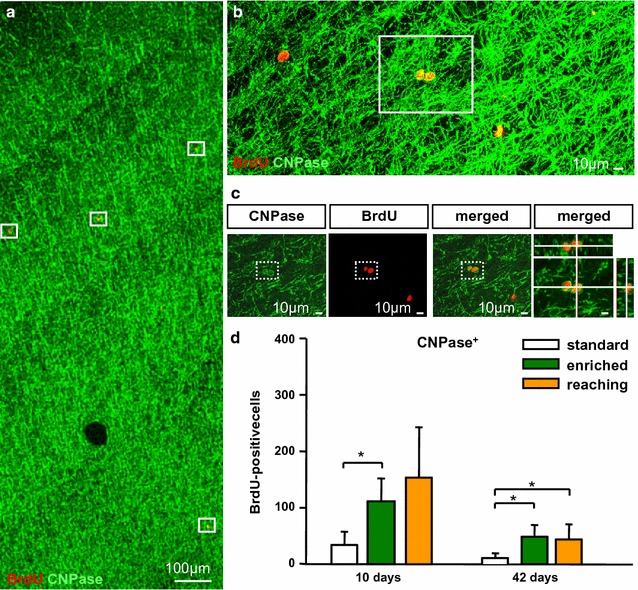
Activity-dependent increase of BrdU^+^CNPase^+^ cells in the sensorimotor cortex. **a** Confocal images of double-labelled sections with antibodies against BrdU (*red*) and CNPase (*green*). **b** Maximum intensity projection showing BrdU^+^ nuclei co-localisation with CNPase. **c** Higher magnification of **(b)** showing single confocal planes by z-stack co-labeling of BrdU^+^ with CNPase^+^. Counted cells were typically surrounded by CNPase expression (in the frame). **d** Graph depicting the number of BrdU^+^CNPase^+^ which increased after enriched environment housing and reaching training at day 42 compared to standard conditions. *Bars* represent mean ± SD. *Asterisks* indicate significant differences (*P* < 0.05). *Scale bars* represent **a** 100 µm, **b**, **c** 10 µm

EE significantly increased the number of newly differentiated oligodendrocytes at day 10 (112 ± 41 cells) and day 42 (49 ± 21 cells) compared to standard conditions (10 days: 34 ± 24 cells; 42 days: 11 ± 8 cells) (Fig. 3d). Moreover, RT slightly increased the number of BrdU^+^CNPase^+^ oligodendrocytes at day 10, and became significant at day 42 (44 ± 27 cells) compared to standard housing. No differences between both activity groups were observed at day 10 and 42 (Fig. 3d). These findings indicate that EE and RT for 6 weeks significantly increase the number of newly differentiated CNPase^+^ oligodendrocytes in the sensorimotor cortex.

**Fig. 4:**
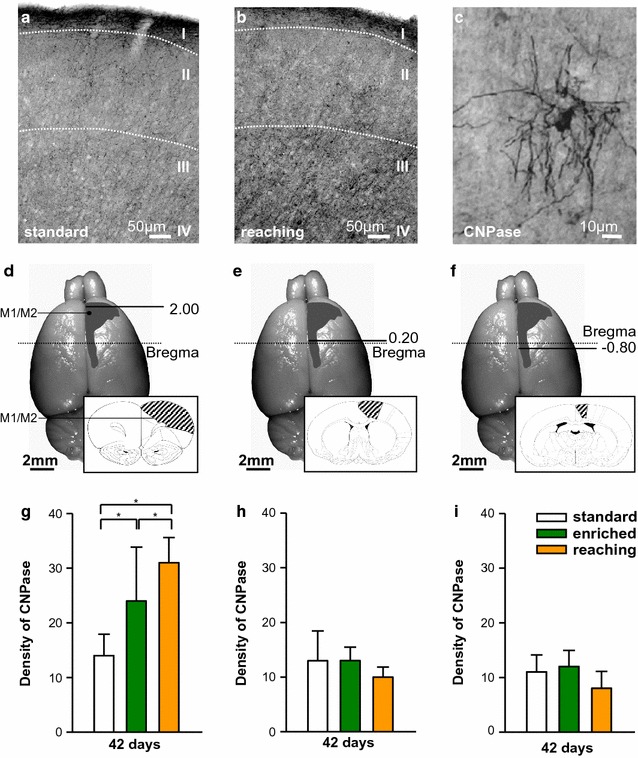
**a**, **b** Peroxidase-stained sections demonstrating CNPase^+^ oligodendrocytes after standard housing (**a**) compared to reaching training (**b**). **c** Higher magnification of CNPase^+^ cells. **d**–**f** Location of the different evaluated regions (Bregma 2.00, 0.20, −0.80) from the motor cortex (M1/M2) marked on the brain surface. The schematic detailed picture shows areas analysed [39]. **g**–**i** Quantification of the optical density of CNPase^+^ cells at Bregma 2.00 (**g**), Bregma 0.20 (**h**) and −0.80 (**i**). Significant differences are observed at Bregma 2.00 between the different activity groups compared to standard housing. *Bars* represent mean ± SD. *Asterisks* indicate significant differences (*P* < 0.05). *Scale bars* represent **a** 50 µm and **b**, **c** 10 µm

In addition to the number of BrdU^+^CNPase^+^ oligodendrocytes, the level of CNPase expression was measured in three different areas (Bregma coordinates: anterior/posterior +2.00, +0.20 and −0.80) at day 42 (Fig. 4a–f). EE (*P* < 0.013) and RT (*P* < 0.038) significantly increased CNPase expression in the frontal cortex (Bregma anterior/posterior: +2.00) which mainly contains the motor cortex, compared to standard housing (Fig. 4g–i). No changes were observed at Bregma anterior/posterior: +0.20 and −0.80 between the different groups.

### Quantification of newly differentiated NG2 and GSTπ expressing oligodendrocytes in the corpus callosum

**Fig. 5:**
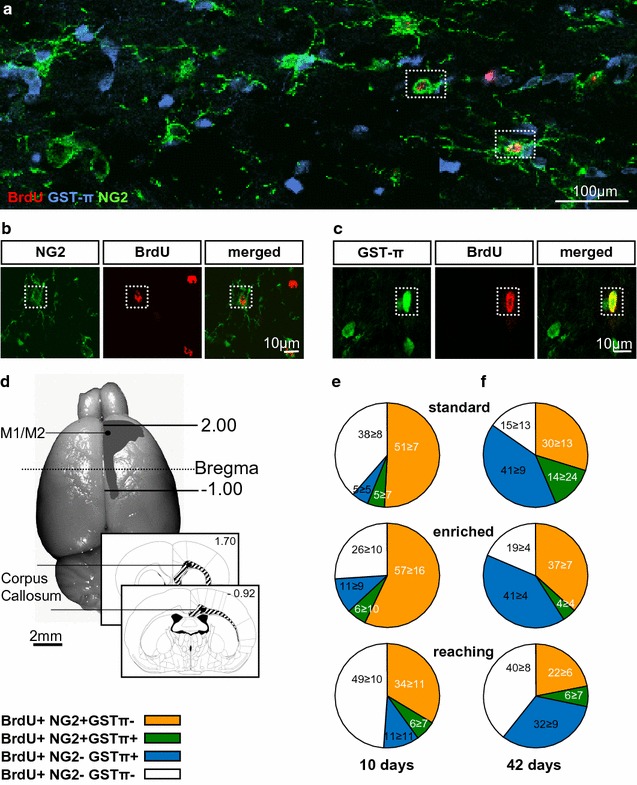
Percentage of distinct oligodendrocyte precursor cells in the corpus callosum of the different experimental groups. **a** Confocal overview image of triple-labelled sections with antibodies against BrdU (*red*), GSTπ (*blue*) and NG2 (*green*). **b** Single confocal planes by z-stack co-labeling of BrdU^+^ with NG2^+^. **c** Single confocal planes by z-stack co-labeling of BrdU^+^ with GSTπ^+^. **d** The schematic detailed picture below shows areas analysed [39]. **e**, **f** The distribution of the BrdU^+^ cells between the different activity conditions at days 10 (**e**) and 42 (**f**). *Scale bars* **a** 100 µm, **b**, **d** 10 µm

After the analysis in the grey matter, we test whether motor activity influences oligodendrocyte precursor cells in the corpus callosum, using BrdU co-labelling (Fig. 5a–d). Almost half of all newly differentiated cells expressed NG2^+^ at day 10 after standard and EE housing. This ratio changed at day 42 as more than 40% of newly differentiated cells expressed only GSTπ^+^ (Fig. 5e/f). The RT showed the same trend to a lower ratio of BrdU^+^NG2^+^ cells at day 10 (*P* = 0.010 to standard housing) and BrdU^+^GSTπ^+^ cells at day 42. Results for the corpus callosum show that the number of NG2^+^ cells declines between 10 and 42 days, whereas the number of GSTπ^+^ cells increases in all groups.

### Quantification of NG2^+^cells expressing doublecortin (DCX) in the cerebral cortex

We determined whether NG2^+^ cells in the cerebral cortex express DCX. Analysis with flow cytometry of freshly dissociated cerebral cortices of 3-month-old rats, confirmed that at least 61 ± 21% of NG2^+^ cells co-expressed DCX (Additional file 3: Fig. S2A) using FACS analysis. Only a small fraction of DCX^+^ cells (42 ± 28%) was found to co-express NG2.

These findings were further validated by immunofluorescence staining. Co-labeling analysis of DCX expression in NG2 cells showed co-localization of DCX in NG2 cells with stellate morphologies in the cerebral cortex (Fig. S2B). Quantitative analysis of histological sections showed that 79 ± 24% of NG2^+^ cells co-labelled with DCX^+^ in all cortical layers. In contrast, 25 ± 6% of the DCX^+^ cell population expressed NG2 (Additional file 3: Fig. S2B). Increased number of DCX^+^ cells co-expressing NG2 was detected with the manual counting after immunofluorescence staining. In addition to protein analysis, we evaluated specific expression of DCX in FACS-sorted, NG2^+^ cells by means of qPCR (Additional file 3: Fig. S2C). The mRNA levels in NG2^+^ sorted cells and hippocampal DCX^+^ were similar. These results demonstrate expression of DCX in NG2^+^ cells. Taken together, we show here that most of NG2^+^ cells co-express DCX in the cerebral cortex.

### Quantification of NG2^+^DCX^+^ cells after EE and RT in the sensorimotor cortex

**Fig. 6:**
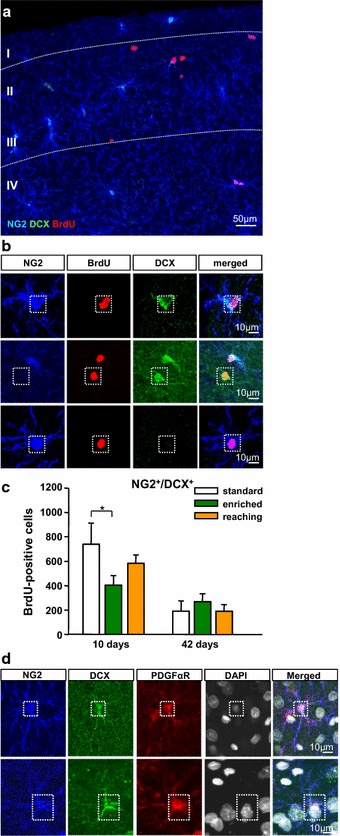
Immunocytochemical quantification of newly differentiated NG2^+^DCX^+^ subpopulation in the sensorimotor cortex of the different experimental groups. **a** Overview of proliferating BrdU^+^ (*red*) NG2^+^ (*blue*) DCX^+^ (*green*) cells at day 10 of the experiment. **b** Confocal images of triple-labelled sections with antibodies against BrdU (*red*) NG2 (*blue*) DCX (*green*) in the sensorimotor cortex. **c** Quantification of BrdU^+^NG2^+^DCX^+^ cells in the different experimental groups at days 10 and 42. **d** Confocal images of quadruple-labelled sections with antibodies against NG2 (*blue*), DCX (*green*), PDGFαR (*red*) and DAPI (*white*). NG2^+^/DCX^+^ cells co-labelled with an antibody against the PDGFα receptor in the adult cerebral cortex. *Error bars* represent S.D. Significant differences (*P* ≤ 0.05) are indicated by an *asterisk*. *Scale bars* **a** 50 µm and **b** 10 µm

We analyzed the effect of motor activity on BrdU^+^NG2^+^DCX^+^ cells in the sensorimotor cortex. Newly differentiated NG2^+^DCX^+^ cells were found in all cortical layers without any preference (Fig. 6a/b). Furthermore, this progenitor subpopulation was significantly reduced by enriched environment housing (404 ± 78 cells/mm^3^, *P* = 0.029) compared to standard housing conditions (738 ± 173 cells/mm^3^) at day 10, whereas specific training of the dominant forelimb (582 ± 69 cells/mm^3^) had no significant effect (Fig. 6c). At day 42, these there were no differences anymore (Fig. 6c). In addition, not all cells expressed both markers; there were some DCX^+^ cells lacking NG2 and NG2^+^cells lacking DCX (Fig. 6b). NG2^+^/DCX^+^ expressing cells were co-labelled for platelet-derived growth factor-alpha receptor (PDGFαR) and were identified as oligodendrocyte precursor cells (Fig. 6d).

## Discussion

The aim of the present study was to analyse the effect of two different motor activity conditions, reaching training (RT) and enriched environment (EE), on differentiation of oligodendrocytes in the sensorimotor cortex and corpus callosum of the adult rat brain. We provide novel data showing that EE and RT act differently on the differentiation process of oligodendrocyte subpopulations in the cerebral cortex and corpus callosum of the rat.

### EE and RT increase the number of newly differentiated oligodendrocytes in the sensorimotor cortex

In accordance with previous studies [2, 5, 9, 21, 40], our findings demonstrate that oligodendrocyte precursors expressing NG2 are the dominant proliferating population in the neocortex of the adult brain. Under standard conditions, in the sensorimotor cortex 79 – 89% of all NG2^+^ cells were newly differentiated NG2^+^ cells, whereas this number was significantly reduced by EE. While BrdU^+^NG2^+^ cells decreased in the sensorimotor cortex of the enriched group, BrdU^+^NG2^+^GSTπ^+^ and BrdU^+^CNPase^+^ oligodendrocyte numbers were increased. These findings are in agreement with a previous study by Simon et al. [46], which described a reduced differentiation of BrdU^+^NG2^+^ cells and a strong increase in the number of BrdU^+^GSTπ^+^ oligodendrocytes in the grey matter after voluntary wheel running. Analysis of proliferation markers Ki67 and BrdU [46] indicates that reduction of BrdU^+^NG2^+^ cell numbers depends on its increased exit from the cell cycle. This, in turn leads to a faster differentiation into mature oligodendrocytes [29, 46]. Our study provides evidence that EE but not RT reduces the number of BrdU^+^NG2^+^ cells. The differential effect of EE and RT on BrdU^+^NG2^+^ cells is possibly associated with different settings of housing and training conditions (comfortable versus stress), the specification of motor activity and learning to the sensorimotor cortex (connectivity or involvement of different brain areas) or direct alterations to the cell (cell cycle regulation, cell death or differentiation). Furthermore, RT increases the number of oligodendrocyte precursors expressing BrdU^+^NG2^+^GSTπ^+^ and also the number of CNPase^+^ oligodendrocytes. In support of our findings, a previous study by Wurm et al. [54] showed a significant increase of newly generated neurons in the dentate gyrus of the hippocampus formation after reaching training, suggesting that reaching training directly influences learning and memory. Taken together these results demonstrate that experiences such as enriched housing, reaching training and wheel running, increase newly differentiated oligodendrocytes in the grey matter. Interestingly, findings from our study are in disagreement with a previous analysis by Ehninger and Kempermann [12] on the effects of enriched environment and voluntary wheel running on basal cell proliferation in the cortex. The study showed a regional and laminar specific regulation of BrdU^+^ cells and described a voluntary wheel running-specific increase of newly generated microglia in the motor cortex, whereas enriched environment housing did not show any particular effects. In addition, they could not detect any motor activity-dependent changes in the number of newly differentiated NG2^+^ cells in the different layers. This discrepancy to our findings might be explained by differences in methodology, since Ehninger and Kempermann [12] used a different experimental design involving BrdU application after 30 days of environmental enrichment housing. Our findings clearly demonstrate that motor activity for 10 days increases the total number of newly differentiated oligodendrocytes (BrdU^+^NG2^+^GSTπ^+^ cells) and CNPase^+^ cells at day 42 in the sensorimotor cortex. Xiao et al. [55] analysed oligodendrocyte differentiation directly after exposure to a complex running wheel. Using in situ hybridization to determine Enpp6 mRNA levels, they analysed early time points after motor skill learning. After complex wheel running, expression of Enpp-6 in immature oligodendrocytes was increased in the subcortical white matter within just 2.5 h and in the motor cortex after 4 h. The block of Enpp-6 by myelin regulatory factor (Myrf) deletion in immature oligodendrocytes impaired motor skill learning in the complex wheel running. These results clearly demonstrate that oligodendrocytes already contribute to learning in early stages of their development. Oligodendrocytes are highly connected to the axons and their metabolism. The development of oligodendrocytes and myelination is regulated by the activity of the axons. Increasing myelination improves the network connectivity at the synaptic level, which could contribute to facilitated learning conditions. Enriched environment housing not only influences the generation of NG2^+^ cells under physiological conditions but also after stroke in remote areas of cortical regions [24, 27], suggesting that EE has a high impact on NG2^+^cells.

The present study also showed a motor activity-dependent increase in the expression of CNPase in the adult sensorimotor cortex. Our findings are in agreement with early studies analysing the glial response to enriched environment in the occipital cortex [1, 10, 23, 48]. In particular, using thionin staining, Diamond et al. [10] found no differences in the number of neurons but a prominent increase in the number of oligodendrocytes as well as astrocytes after 80 days of enriched housing. Szeligo and Leblond [48] and Katz and Davies [23] confirmed the activity-induced increase in the number of oligodendrocytes in the occipital cortex after 30 or 80 days enriched environment housing. Based on their results, these authors suggested that the overall sensory motor stimulation influences the activity of neurons in the occipital cortex leading to an increase of the number of oligodendrocytes. In addition, rats reared in a complex enriched environment showed significantly more oligodendrocytes in the visual cortex [47]. Interestingly, social isolation of young mice (sensory deprivation) decreases the number of oligodendrocytes in the prefrontal cortex [34], further supporting previous findings.

We hereby additionally demonstrate that CNPase expression in the sensorimotor cortex responds to external stimulation such as EE and RT. Therefore, we suggest that CNPase expression in oligodendrocytes might contribute to the activity-dependent remodelling of synaptic connections in the adult cortex.

### The percentage distribution of different oligodendrocyte subtypes is not changed by motor activity in the corpus callosum

The distribution of distinct oligodendrocyte subtypes was analysed in the corpus callosum at day 10 and day 42. We found a strong decrease in the percentage of proliferating NG2^+^ cells between day 10 and day 42. This was observed in all groups and reflects the differentiation of NG2^+^ cells. Sampaio-Baptista and colleagues [42] showed an increase of mature oligodendrocytes analysed by MBP expression after reaching training. They further found a positive correlation between learning rate and diffusion MRI fractional anisotropy (FA) in the contralateral hemisphere of the trained paw. Interestingly, human motor learning and training such as those undertaken by professional musicians modify the white matter showing an enlarged anterior corpus callosum compared to controls [15, 38, 43]. Scholz et al. [44] analysed the effect of 6 weeks of juggling training on adult human white matter and detected a significant increase of diffusion MRI fractional anisotropy in the right posterior intraparietal sulcus for a period of at least 4 weeks when compared to controls.

### Enriched environment decreases the number of DCX-expressing NG2 cells in the sensorimotor cortex

Our analysis concentrated on a subpopulation of NG2^+^ cells expressing DCX in the adult rat sensorimotor cortex. Here, using immunohistochemistry and flow-cytometry-based analysis we detected a high fraction of NG2^+^DCX^+^ cells in the cerebral cortex. Numbers of cells as determined by flow cytometry were higher compared with numbers from immunohistochemical analysis. The difference in the number of cells between both methods is possibly due to higher sensitivity of flow cytometry-based analyses compared with in situ staining methods. DCX has been identified as a micotubule-associated protein important for neuroblast migration in the developing and adult brain. At this stage, DCX is involved in microtubulin stabilization, nuclear translocation and growth cone dynamics [14, 17, 26, 53]. Interestingly, Hughes et al. [21] demonstrated that NG2^+^ cells migrate continuously through the adult cortex. To what extent DCX expression in NG2^+^ cells is necessary for NG2 motility is still unclear and needs to be further investigated. In contrast to the findings for NG2^+^ cells, the number of DCX^+^ cells expressing NG2 is rather low. Expression of other glial markers such as GFAP in DCX^+^ cells has already been described for humans, primates and mice [3, 28]. Next, we analysed to what extent enriched environment and skilled reaching training influenced newly differentiated NG2^+^DCX^+^ cells in the sensorimotor cortex. Interestingly, EE decreased the subpopulation of NG2^+^DCX^+^ cells. In support of our findings, several studies described the co-expression of NG2 and DCX in the adult brain [13, 18, 49, 50]. Ehninger et al. [13] detected a large fraction of BrdU^+^NG2^+^DCX^+^ cells 1 day and 4 weeks after the last BrdU injection in the adult amygdala. Guo et al. [18] reported that the adult piriform cortex contains a high number of DCX-expressing cells with different DCX expression levels, which was additionally described by Walker et al. [51]. Our results demonstrate that unspecific training like EE differentially influences newly differentiating NG2^+^DCX^+^ subpopulation compared to specific forelimb training. The possible mechanisms underlying the effect of EE on the number of NG2^+^DCX^+^ cells in the sensorimotor cortex are still not clear.

In the present study we detected a reduction in brain volume restricted to Bregma 2.7–5.3 mm after enriched environment and standard housing conditions compared to reaching training. These results give some evidence that EE and RT differentially influence the brain volume, which was previously described using motor map expression [58]. To validate these findings more sensitive methods such as MRT must be used to get insights into changes in the whole brain volume due to experience.

### Possible mechanisms and functional consequences

The present study provides evidence that enriched environment and skilled reaching training can regulate oligodendrocyte differentiation in the sensorimotor cortex. Previous studies have shown that activity, such as running or individual learning of sensorimotor skills, can induce the generation of oligodendrocytes. Possible mechanisms involved in increased oligodendrocyte genesis might include altered cell cycle dynamics, modulation of growth factors or increasing axon stimulation by neuronal activity [16, 30, 46]. The mechanisms responsible for the increased number of oligodendrocytes in the sensorimotor cortex after enriched environment housing and skilled reaching training, are totally unclear and need further investigations. The pivotal role of oligodendrocytes in the adult cortex is the myelination of axons within the neuronal network. Myelinating oligodendrocytes thereby control the speed of impulse conduction within these networks and adjust the synchrony of impulse traffic between distant cortical regions. Increased activity of the neural circuit might stimulate myelination of the axons and make the network more efficient. The functional relevance of newly differentiating oligodendrocytes has already been shown by McKenzie et al. [35] and Xiao et al. [55]. Both studies showed that in mice, differentiation of new oligodendrocytes is indispensable to learn the complex wheel paradigm. It is very likely that these new oligodendrocytes contribute to the activity-dependent remodelling of synaptic connections in the adult cortex.

## Conclusion

Taken together, the present study clearly demonstrates that two different types of motor activities, such as specific motor skill training and unspecific training in an enriched environment, differentially influence the differentiation of specific oligodendrocyte subpopulations in the adult sensorimotor cortex.

## Methods

### Experimental design

In the current study, enriched environment was used to promote forelimb unspecific motor activity and was compared to skilled reaching training as a specific training of the dominant forelimb (Fig. 1a). Environmental enrichment is an experimental paradigm that offers physical and social stimulation to the animal. Skilled reaching training is characterised by repetitive grasping of pellets with the dominant forelimb. Forty-one male Wistar rats (250–270 g, 10–12 weeks) were randomly assigned to three experimental groups (Fig. 1). One group (n = 13) was housed in an enriched environment cage (6–7 animals/cage, 85 cm × 75 cm × 40 cm) containing several stimuli comprising objects such as toys, climbing boards, chains, ladders and tunnels, but no running wheel. The configuration of the objects was changed on a daily basis. A second group of animals lived under standard conditions and received daily reaching training of the dominant forelimb in a Plexiglas reaching box (n = 15) beginning at day 1 of the experiment. Forelimb preference was assessed before the experiment began [54]. The rats started with 50 pellets daily in the first week, which was increased to 100 pellets per day in the second week (Dustless Precision Pellets 45 mg, BioServ, USA). A third group (control group) (n = 13) remained in the normal standard housing conditions without stimulated motor activity (4 animals/cage; 54 cm × 38 cm × 19 cm) during the whole experiment. All animals were food deprived to 95% of their initial body weight. All data were acquired by independent observers unaware of the experimental conditions. All experimental procedures were approved by the German Animal Care and Use Committee in accordance with the European Directives. All rats used in the study were bred in our animal facility at the Jena University Hospital, and were originally provided by Harlan-Winkelmann GmbH in Germany.

### 5-Bromo-2′deoxyuridine (BrdU) injections

The thymidine analogue BrdU was used to trace proliferating cells in the sensorimotor cortex. Beginning at day 2 after animals were separated into 3 groups, intraperitoneal injections of Bromodeoxyuridine (BrdU, 50 mg/ kg, Sigma-Aldrich, Taufkirchen, Germany) were applied for five consecutive days to all rats. Animals were allowed to survive for either 10 or 42 days (Fig. 1).

### Tissue preparation and immunohistochemistry

Animals were anesthetized and transcardially perfused with 4% paraformaldehyde in 0.1 M phosphate buffer. Brains were removed and postfixed for 24 h, cryoprotected with 10% for one day followed by 30% sucrose. Consecutive Sections (40 µm) were cut using a freezing microtome and stored at −20 °C.

Immunocytochemistry was performed on free floating sections. To allow for NG2, BrdU or 2′,3′-cyclic nucleotide 3′-phosphohydrolase (CNPase) detection, sections were treated with 0.6% H_2_O_2_ in Tris-buffed saline (TBS) for 30 min followed by several rinses in Tris-buffered saline containing 0.1% Triton X-100 (Tris-Triton). Further, sections were incubated with primary antibodies rabbit monoclonal anti-NG2 (1:500; Chemicon, CA, USA, Cat.No. AB5320), rat monoclonal anti-BrdU (1:500; Immunologicals Direct-Oxford Biotechnology, Oxfordshire, UK, Cat.No. OBT0030CX) or mouse monoclonal anti-CNPase (1:500; Abcam, Cambridge, UK, Cat.No. AB6319) in TBS-Triton with 3% normal donkey serum overnight at 4 °C. After 24 h, sections were rinsed with Tris-buffered saline and incubated in biotinylated donkey anti-rabbit, anti-rat or anti-mouse antisera (1:500, Jackson Immunoresearch, West Grove, PA) for 2 h at room temperature. After several rinsing steps, the avidin–biotin-peroxidase complex (Vector Laboratories, Burlingham, CA, USA) was incubated for 60 min at room temperature. Labelled cells were visualized using 3.3-diaminobenzidine solution (0.25 mg/ml, Sigma Aldrich, Munich, Germany) as chromogen. Sections were washed, mounted, and covered.

Immunofluorescent double and triple labelling was performed in the same way as described previously for immunoperoxidase staining but without the 0.6% H_2_O_2_ washing step. Sections were incubated for 24 h with the primary antibodies rat anti-BrdU (1:500; Immunologicals Direct-Oxford Biotechnology, Oxfordshire, UK, Cat.No. OBT0030CX); goat anti-DCX (1:500; Santa Cruz, CA, USA, Cat.No. SC-8066), rabbit anti-NG2 (1:500; Chemicon, CA, USA, Cat.No. AB5320); mouse anti-NG2 (1:500; Millipore, CA, USA, Cat.No. MAB5384); mouse anti-GSTπ (1:500; Bioscience, CA, USA, Cat.No. 610718), rabbit anti-PDGFRa (1:500; Santa Cruz, CA, USA, Cat.No. SC-338) and mouse anti CNPase (1:500; Abcam, Cambridge, UK, Cat.No. AB6319). The following secondary antibodies were used: Rhodamine anti-rat (1:500, Dianova, Hamburg, Germany, Cat.No. 712-295-150), Alexa Fluor 647 anti-mouse (1:500, Dianova, Hamburg, Germany Cat.No. 715-605-151), Alexa Fluor 488 anti-rabbit (1:500, Molecular Probes, Leiden, Netherlands, Cat.No. A21206), Alexa Fluor 647 anti-rabbit (1:500, Dianova, Hamburg, Germany, Cat.No. 711-606-152) and Alexa Fluor 488 anti-goat (1:500, Molecular Probes, Leiden, Netherlands, Cat.No. A11055).

### Brain volumetry

Brain volume was measured using a charge-coupled device (CCD) camera and National Institute of Health (NIH), USA Image (software package). The area of the hemispheres (mm^2^) was analysed by tracing these regions on the computer screen for every 8th Section (40 µm) from 2.7 mm through −5.3 mm to Bregma [39]. Brain volume (mm^3^) was calculated by integrating the measured area with the section interval thickness (320 µm).

### Cell quantification

To quantify the CNPase^+^ oligodendrocytes, light microscope images of the sensorimotor cortex of three different areas (coordinates from Bregma were anterior/posterior +2.00, +0.20 and −0.80) were digitized (×125) using a CCD color video camera (Axiocam HR, Carl Zeiss, Jena, Germany) under standardized conditions. The mean optical density of CNPase immunoreactivity was quantified by binary images using image analysis program with density values ranging between “white” (0) and “black” (255) (Scion Image 6.21; National Institutes of Health, Bethesda, MD).

NG2^+^ cells were stereologically quantified in the forelimb sensorimotor cortex (2.0 mm anterior and −1 mm posterior to Bregma according to Paxinos [39]) using the optical fractionator method and a semiautomatic stereological system (StereoInvestigator, MicroBrightField, Colchester, USA). The total number of NG2^+^ cells was counted on every 12th section of the peroxidase-stained coronal slices in each rat. To trace the sensorimotor cortex, we used a 5× objective and counted all positive cells using a 63× objective. The number of NG2 labelled cells was related to 1/mm^3^ sectional volume to estimate the total number of NG2^+^ cells per rat.

The quantification of BrdU^+^ cells in the sensorimotor cortex was processed by using a light microscope (Axiocam HR, Carl Zeiss, Jena, Germany) with a 40 × objective. Every 6th section (40 µm) was counted (2.0 mm anterior and −1 mm posterior to Bregma according to Paxinos [39]) and multiplied by the factor 6 to obtain the total number of BrdU^+^ cells for each rat brain.

Phenotype analysis of BrdU^+^ cells in the sensorimotor cortex and corpus callosum (Bregma: +1.70 to −1.00) was determined on double- or triple-immunofluorescence sections using the confocal laser scanning microscope (Zeiss, LSM 710, Jena, Germany). The phenotype of 50–60 BrdU-labelled cells for each rat was confirmed by z-stacks, allowing the exact assignment of the nucleus to the different antigens. The absolute number of cells with the different phenotypes in the sensorimotor cortex was calculated per animal by multiplying the total number of BrdU^+^ cells (peroxidase method) with the percentage of co-labelled cells per antigen. The ratio of the different phenotypes was determined in the corpus callosum and sensorimotor cortex.

The same counting procedure was used for the NG2^+^DCX^+^ cells in the cerebral cortex, multiplying the total number of NG2^+^ cells (peroxidase method) with the percentage of co-labelled DCX^+^ cells.

All cell quantifications and phenotype analyses were performed on the contralateral side of the dominant forelimb of the reaching group.

### Flow Cytometry

To characterize and quantify the NG2-DCX co-expression, a flow cytometry-based analysis was performed. Neocortical brains from 12-week old rats (n = 10, all layers excluding subcortical structures) were removed, transferred to ice-cold HBSS and placed in a pre-chilled Petri-dish. Tissue was triturated using fire-polished Pasteur pipettes of different diameters starting with the larger size. Disaggregated tissue was centrifuged and incubated in PBS, 10% normal donkey serum (NDS) and 1% sodium azide, followed by 3% bovine serum (BSA), 3% NDS and 0.2% Tween20, fixed by 4% PFA and washed in 0.2% Tween20. Further, cells were incubated in 0.2% saponin, washed in 0.2% Tween20, followed by blocking in 5% BSA and washed again in 0.2% Tween20. After a new centrifugation step, the cell pellet was incubated with the following primary antibodies: goat anti-DCX (1:100; Santa Cruz, CA, USA) and rabbit anti-NG2 (1:100; Chemicon, CA, USA) in 3% BSA, 3% NDS and 0.2% Tween20 overnight at 4 °C. The cell suspension was centrifuged and the pellet was rinsed several times in PBS and 3% BSA, 3% NDS, 0.2% Tween20 and 10% NDS with 1% sodium azide and centrifuged again between the washing steps. Cell were then incubated with secondary antibodies for 2 h at room temperature in 3% BSA, 3% NDS and 0.2% Tween20. The following secondary antibodies were used: Rhodamine anti-goat (1:250, Dianova, Hamburg, Germany) and Alexa Fluor 488 anti-rabbit (1: 500, Molecular Probes, Leiden, Netherlands). After centrifugation, cells were washed several times with PBS followed by incubation in 3% BSA and 1% Sodium azide. All centrifugation steps were performed at 2000 rpm for 5 min. Flow cytometry was performed using a FACSCalibur™ flow cytometer (BD Biosciences, Heidelberg, Germany). Single positive and isotype stains were used as controls for compensation and gating thresholds. Data were analyzed using Cell Quest Pro™ (BD Bioscience, Heidelberg, Germany).

### Fluorescence-activated cell sorting and qPCR

Isolation of NG2^+^ cells was carried out by fluorescence activated cell sorting (FACS) using a MoFlo cell sorter (Beckman Coulter, Krefeld, Germany). Brains were removed from 3 month-old Wistar rats (n = 6). The brain dissociation and staining procedure was performed as described above without any fixation steps. Cell suspensions were initially incubated with the primary antibody rabbit anti-NG2 (1:100; Chemicon, CA, USA) followed by secondary antibody Alexa Fluor 488 anti-rabbit (1:250, Molecular Probes, Leiden, Netherlands).

Immediately following FACS, RNA isolation from the NG2 sorted fraction, reverse transcription and qPCR was undertaken as previously described by [45]. Specific primers for doublecortin (forward: ggccaaactggtgtgagatt; reverse: ccccaggtatgccagataga) were used.

### Statistical analysis

Analysis of cell counts and phenotype assessment was firstly tested for one-way analysis of variance using the Kruskal–Wallis test, followed by a Mann–Whitney-*U*-test. All statistical analyses were performed using SPSS for Windows 11.5 (Standard Version). All cell counts and phenotype analysis are expressed as mean ± S.D. Significant differences were assumed at a level of *P* < 0.05.

## Additional files

**Additional file 1:** **Table S1.** Quantification of BrdU-positive cells and colocalization with NG2 in the sensorimotor cortex. Mean ± SD. Statistical significance between the activity groups and standard animals is indicated by an asterisk (P ≤ 0.05).

**Additional file 2:** **Figure S1.** Percentage distribution of distinct proliferating oligodendrocyte precursor cells from the standard, enriched and reaching group in the adult sensorimotor cortex. At day 10 the enriched group showed a higher percentage of NG2^+^GSTπ^+^ cells compared to standard and reaching conditions.

**Additional file 3:** **Figure S2.** Characterisation of NG2^+^ and DCX^+^ cells by flow cytometry analysis, immunohistochemical analysis and qPCR. (**A**) The percentage of NG2^+^DCX^+^ cells was about 61% in the adult rat cortex after flow cytometry analysis. (**B**) Compared to flow cytometry analysis, about 79% of NG2^+^ cells were co-labelled against DCX. (**C**) DCX expression pattern flow cytometry-sorted NG2 cells identified by qPCR. MW, molecular weight; Ctrl, control for rat DCX Primer. Error bars represent S.D.

## Abbrevations

RT: reaching training of the dominant forelimb
EE: enriched environment
BrdU: 5-bromo-2′deoxyuridine
CNPase: 2′,3′-cyclic nucleotide 3′-phosphohydrolase
NG2: neural/glial antigen 2
GSTπ: glutathione S-transferase π
DCX: doublecortin
PDGFαR: platelet-derived growth factor-alpha receptor
Enpp6: ectonucleotide pyrophosphatase/phosphodiesterase 6
Myrf: myelin regulatory factor
FA: fractional anisotropy
CCD: charge-coupled device camera
FACS: fluorescence activated cell sorting
