# Supplementary material for: Effect of skilled reaching training and enriched environment on generation of oligodendrocytes in the adult sensorimotor cortex and corpus callosum
Source: BMC Neurosci. 2017 Mar 9;18:31. doi: 10.1186/s12868-017-0347-2 (PMC5345235; doi:10.1186/s12868-017-0347-2)
Supplement: Supplementary file 1 — Additional file 1: Table S1. Quantification of BrdU-positive cells and colocalization with NG2 in the sensorimotor cortex. Mean ± SD. Statistical significance between the activity groups and standard animals is indicated by an asterisk (P ≤ 0.05). [file 12868_2017_347_MOESM1_ESM.docx]

|  |  | **10 days** | | |  | **42 days** | | |  |
| --- | --- | --- | --- | --- | --- | --- | --- | --- | --- |
|  |  |  |  |  |  |  |  |  |  |
|  | |  |  |  |  |  |  |  |  |
| **standard** | BrdU^+^ (total) | 830 | ± | 179 |  | 337 | ± | 123 |  |
|  |  |  |  |  |  |  |  |  |  |
|  | BrdU^+^NG2^+^ (%) | 89 | ± | 4.63 |  | 79 | ± | 19.79 |  |
|  |  |  |  |  |  |  |  |  |  |
|  |  |  |  |  |  |  |  |  |  |
|  |  |  |  |  |  |  |  |  |  |
| **enriched** | BrdU^+^ (total) | 577 | ± | 183 * |  | 407 | ± | 87 |  |
|  |  |  |  |  |  |  |  |  |  |
|  | BrdU^+^NG2^+^ (%) | 75 | ± | 9.14 * |  | 76 | ± | 9.84 |  |
|  |  |  |  |  |  |  |  |  |  |
|  |  |  |  |  |  |  |  |  |  |
|  |  |  |  |  |  |  |  |  |  |
|  |  |  |  |  |  |  |  |  |  |
| **reaching** | BrdU^+^ (total) | 904 | ± | 188 |  | 276 | ± | 66 |  |
|  | BrdU^+^NG2^+^ (%) | 80 | ± | 14.97 |  | 73 | ± | 12.60 |  |
|  |  |  |  |  |  |  |  |  |  |
|  |  |  |  |  |  |  |  |  |  |
