# Supplementary material for: Effect of skilled reaching training and enriched environment on generation of oligodendrocytes in the adult sensorimotor cortex and corpus callosum
Source: BMC Neurosci. 2017 Mar 9;18:31. doi: 10.1186/s12868-017-0347-2 (PMC5345235; doi:10.1186/s12868-017-0347-2)
Supplement: Supplementary file 2 — Additional file 2: Figure S1. Percentage distribution of distinct proliferating oligodendrocyte precursor cells from the standard, enriched and reaching group in the adult sensorimotor cortex. At day 10 the enriched group showed a higher percentage of NG2+GSTπ+ cells compared to standard and reaching conditions. [file 12868_2017_347_MOESM2_ESM.ppt]

## Slide 1
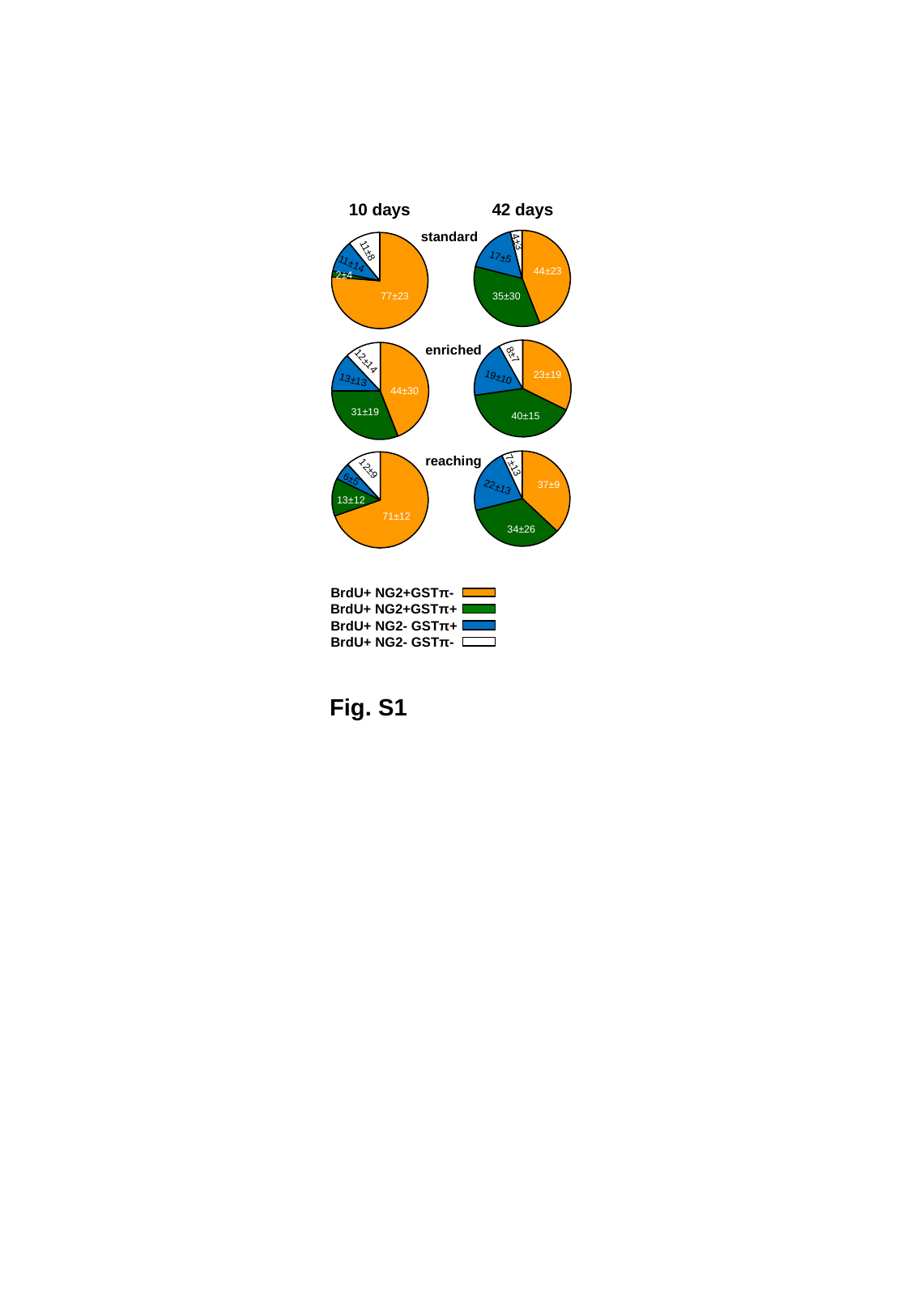

10 days
42 days
standard
4±3
11±8
17±5
11±14
44±23
2±4
77±23
35±30
enriched
8±7
12±14
23±19
19±10
13±13
44±30
31±19
40±15
reaching
7±13
12±9
6±5
37±9
22±13
13±12
71±12
34±26
BrdU+ NG2+GSTπ-
BrdU+ NG2+GSTπ+
BrdU+ NG2- GSTπ+
BrdU+ NG2- GSTπ-
Fig. S1
