# Supplementary material for: Effect of skilled reaching training and enriched environment on generation of oligodendrocytes in the adult sensorimotor cortex and corpus callosum
Source: BMC Neurosci. 2017 Mar 9;18:31. doi: 10.1186/s12868-017-0347-2 (PMC5345235; doi:10.1186/s12868-017-0347-2)
Supplement: Supplementary file 3 — Additional file 3: Figure S2. Characterisation of NG2+ and DCX+ cells by flow cytometry analysis, immunohistochemical analysis and qPCR. (A) The percentage of NG2+DCX+ cells was about 61% in the adult rat cortex after flow cytometry analysis. (B) Compared to flow cytometry analysis, about 79% of NG2+ cells were co-labelled against DCX. (C) DCX expression pattern flow cytometry-sorted NG2 cells identified by qPCR. MW, molecular weight; Ctrl, control for rat DCX Primer. Error bars represent S.D. [file 12868_2017_347_MOESM3_ESM.ppt]

## Slide 1
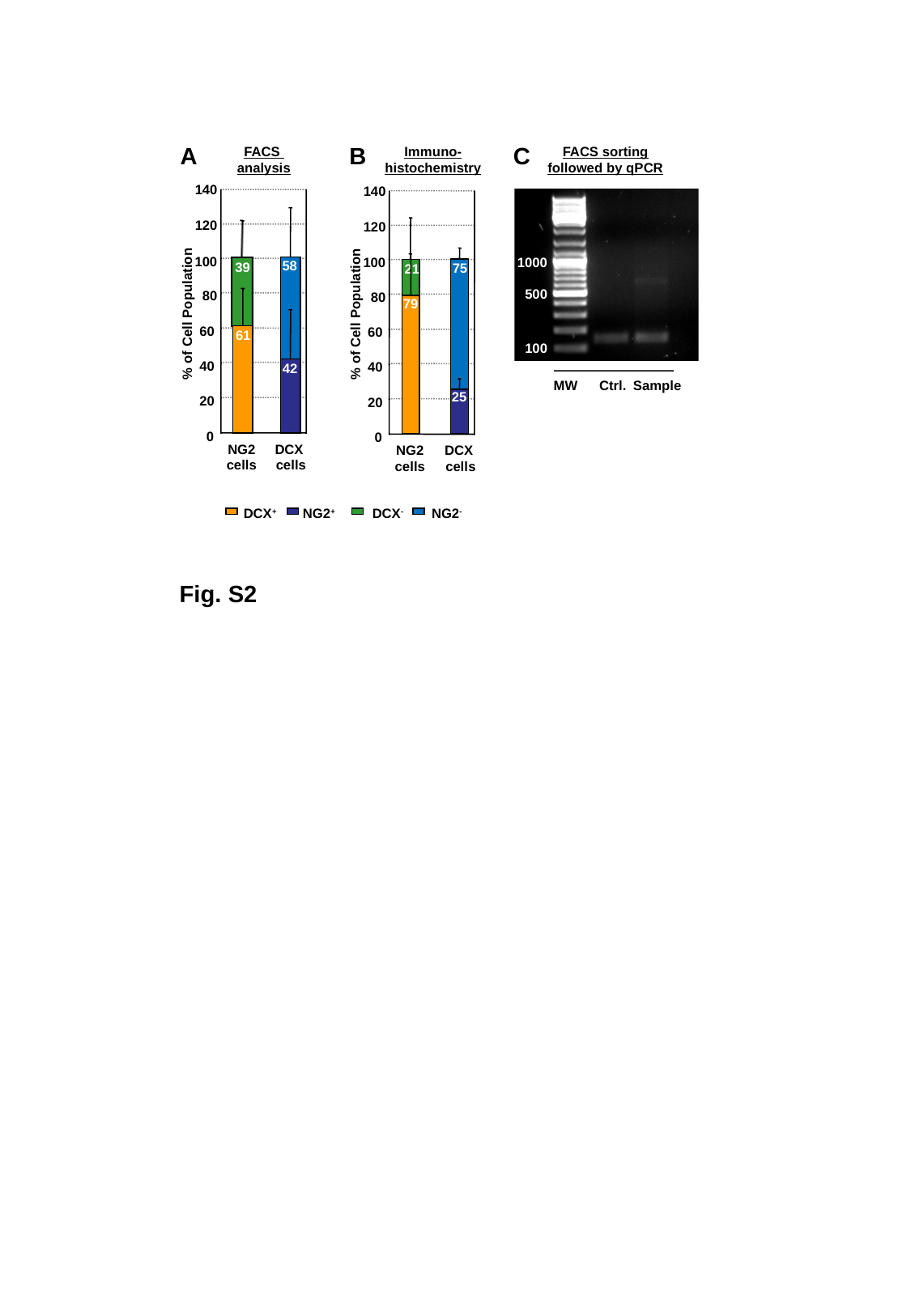

A
B
C
FACS
analysis
Immuno-histochemistry
FACS sorting followed by qPCR
140
140
120
120
58
39
100
75
1000
100
21
500
80
80
79
% of Cell Population
% of Cell Population
61
60
60
100
42
40
40
MW
Ctrl.
Sample
25
20
20
0
0
NG2 cells
DCX
cells
NG2 cells
DCX
cells
DCX+
NG2+
DCX-
NG2-
Fig. S2
